# The benefit of combining a deep neural network architecture with ideal ratio mask estimation in computational speech segregation to improve speech intelligibility

**DOI:** 10.1371/journal.pone.0196924

**Published:** 2018-05-15

**Authors:** Thomas Bentsen, Tobias May, Abigail A. Kressner, Torsten Dau

**Affiliations:** Hearing Systems Group, Department of Electrical Engineering, Technical University of Denmark, Kgs. Lyngby, Denmark; Universidad de Salamanca, SPAIN

## Abstract

Computational speech segregation attempts to automatically separate speech from noise. This is challenging in conditions with interfering talkers and low signal-to-noise ratios. Recent approaches have adopted deep neural networks and successfully demonstrated speech intelligibility improvements. A selection of components may be responsible for the success with these state-of-the-art approaches: the system architecture, a time frame concatenation technique and the learning objective. The aim of this study was to explore the roles and the relative contributions of these components by measuring speech intelligibility in normal-hearing listeners. A substantial improvement of 25.4 percentage points in speech intelligibility scores was found going from a subband-based architecture, in which a Gaussian Mixture Model-based classifier predicts the distributions of speech and noise for each frequency channel, to a state-of-the-art deep neural network-based architecture. Another improvement of 13.9 percentage points was obtained by changing the learning objective from the ideal binary mask, in which individual time-frequency units are labeled as either speech- or noise-dominated, to the ideal ratio mask, where the units are assigned a continuous value between zero and one. Therefore, both components play significant roles and by combining them, speech intelligibility improvements were obtained in a six-talker condition at a low signal-to-noise ratio.

## Introduction

Computational speech segregation attempts to automatically separate speech from interfering noise. This is particularly challenging in single-channel recordings where a speech signal is corrupted by competing talkers and the signal-to-noise ratio (SNR) is low. It has been suggested to exploit *a priori* knowledge about the speech signal and the interfering noise by constructing an ideal binary mask (IBM) [1]. Specifically, the IBM is derived by comparing the SNRs in individual time-frequency (T-F) units to a local criterion (LC). The resulting IBM consists of binary values where T-F units with SNRs exceeding the LC are considered to be speech-dominated and labeled one, whereas T-F units with SNR below the LC are considered to be noise-dominated and are labeled zero. However, since the IBM is unavailable in realistic scenarios, the challenge in computational speech segregation is to estimate the IBM from the noisy speech. Typically, computational speech segregation systems consist of an acoustic feature extraction stage combined with a classification stage where the T-F units are separated into speech-dominated and noise-dominated units in the estimated mask.

In many studies, objective measures have been used to optimize the performance of computational speech segregation systems during the development stage. One commonly used objective measure has been the H-FA rate, which calculates the difference between the percentage of correctly classified speech-dominated T-F units (hit rate, H) and the percentage of incorrectly classified noise-dominated T-F units (false alarm rate, FA) [2–8]. Another commonly used objective measure has been the short-term objective intelligibility (STOI) [9–12]. However, both objective measures have limitations in predicting speech intelligibility. This has been observed with configurations in which the IBM has been degraded with different mask errors [13], and with computational speech segregation systems for noise reduction [14, 15]. Measuring speech intelligibility in listeners is therefore important to properly evaluate changes introduced in a speech segregation system.

Recent approaches in computational speech segregation have considered systems in which the T-F units are predicted by deep neural networks (DNNs). With these *state-of-the-art approaches*, measured speech intelligibility improvements have been demonstrated in various adverse conditions [16–19]. A selection of components may be responsible for the success: the system architecture, a time frame concatenation technique and the learning objective.

First, the system architecture is different than in previously used approaches. In the state-of-the-art approaches, the features are extracted per frequency channel and subsequently stacked across frequency. The T-F units in the estimated mask are then predicted simultaneously across all frequency channels by the DNN. This has consequences for how the context, i.e. the spectro-temporal regions in the estimated mask where speech-dominated T-F units tend to cluster, is exploited by the system. By predicting the T-F units simultaneously across all frequency channels, the state-of-the-art approaches therefore exploit the spectral context in a broadband manner. In previously used approaches, a classifier has been employed per frequency channel (i.e., a subband classifier) in the speech segregation system. These subband classifiers have been implemented with either Gaussian mixture models (GMMs) [2], support vector machines (SVMs) [3, 4] or DNNs [20]. In such a subband-based system, the spectral context has been exploited across neighboring subbands by, for example, including delta features which can capture spectral feature variations across neighboring frequency channels [2, 8, 21].

Secondly, state-of-the-art approaches often exploit temporal context by concatenating extracted feature vectors across a predefined number of time frames [11, 12, 17]. Past and future time frames have both been considered. Improvements in objective measures with time frame concatenation have been reported [11]. However, the effect of employing a time frame concatenation technique on measured speech intelligibility is currently unknown.

Thirdly, state-of-the-art approaches use the ideal ratio mask (IRM) as the learning objective instead of the IBM [16–19, 22]. In the IRM, the mask value is a continuous gain between zero and one and computed according to the *a priori* SNR of the considered T-F unit [11, 23–25]. Therefore, the IRM is similar to an ideal Wiener filter [25]. The perceptual effect of applying IBMs versus IRMs has been investigated in terms of speech quality [26]. A higher sound quality rating with lower noise annoyance and a larger degree of speech naturalness were observed when using IRMs compared to IBMs. Additionally, continuous versus binary gain functions were compared in the framework of minimum mean-squared error (MMSE)-based noise reduction algorithms [27]. It was shown that the continuous gain function outperformed the binary gain function in terms of measured speech intelligibility scores. Furthermore, a larger STOI improvement relative to noisy speech was found with IRM estimation in DNN-based systems compared to IBM estimation [11, 12]. Despite these observations, none of the state-of-the-art approaches has actually demonstrated measured speech intelligibility improvements with IRM estimation over IBM estimation in an otherwise identical system. Furthermore, it is unclear how much IRM estimation contributes to the success of state-of-the-art approaches, especially in comparison to the other components.

The aim of the present study was to explore the roles and the relative contributions of these components within state-of-the-art computational speech segregation by measuring speech intelligibility in normal-hearing (NH) listeners at a low SNR. Specifically, a broadband DNN-based system was compared with a corresponding subband-based system. The subband-based system employed a GMM classifier per frequency channel using delta features across subbands to exploit the spectral context. To exploit temporal context in the DNN-based system, time frame concatenation was either included or excluded. Moreover, the effect of IRM estimation versus IBM estimation was studied in the DNN-based system. To create as fair of a comparison between the different systems as possible, the DNN-based system and the subband GMM-based system considered the same features, and were both trained using the same amount of training data. Therefore, the considered systems were not necessarily designed to maximize the measured speech intelligibility, but instead are designed to be able to systematically compare each of the different components.

## Methods

### Feature extraction

Noisy speech was sampled at a rate of 16 kHz and decomposed into *K* = 31 frequency channels by employing an all-pole version of the gammatone filterbank [28], whose center frequencies were equally spaced on the equivalent rectangular bandwidth (ERB) scale between 80 and 7642 Hz. Previous studies [2, 7, 8] successfully exploited modulations in the speech and the interferer by extracting amplitude modulation spectrogram (AMS) features [29, 30]. To derive the AMS features in each frequency channel (subband), the envelope was extracted by half-wave rectification and low-pass filtering with a cutoff frequency of 1 kHz. Then, each envelope was normalized by its median computed over the entire envelope signal. These normalized envelopes were then processed by a modulation filterbank that consisted of one first-order low-pass and five band-pass filters with logarithmically spaced center frequencies and a constant Q-factor of 1. The cutoff frequency of the modulation low-pass filter was set to the inverse of the window duration to ensure that at least one full period of the modulation frequency was included in the window [8]. Using time frames of 32 ms with 75% overlap (i.e., a 8 ms frame shift) resulted in a cutoff frequency of 32 Hz. The root mean square (RMS) value of each modulation filter was then calculated across each time frame.

### The DNN-based system

**Fig 1:**
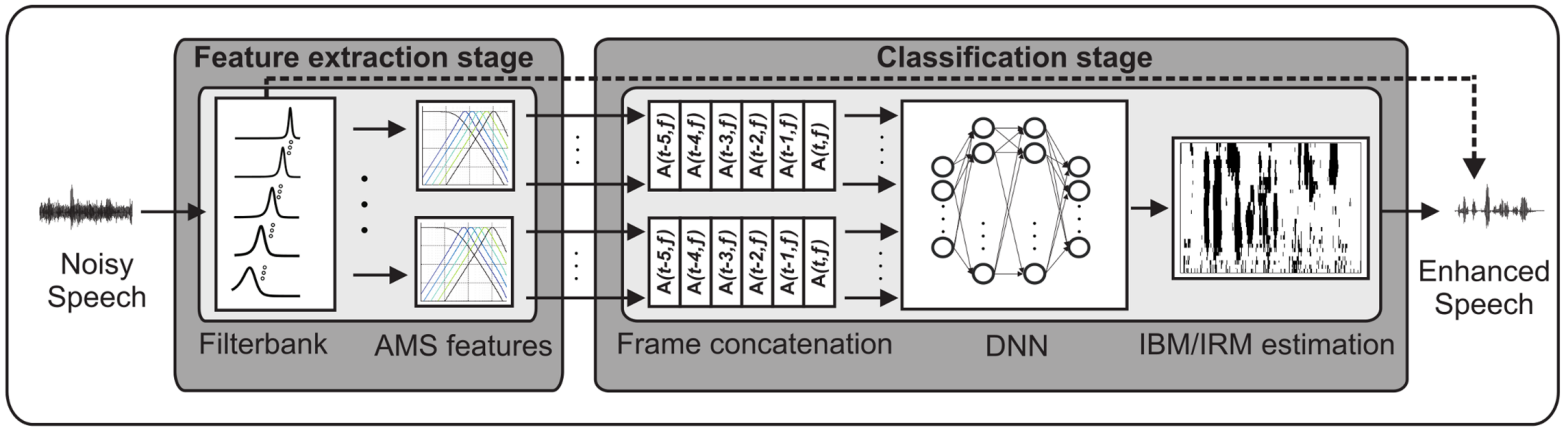
The DNN-based system. Noisy speech was decomposed by a gammatone filterbank and AMS features were extracted per subband. The AMS features were fed into an DNN with two hidden layers of 128 nodes each. The system estimated a time-frequency mask (either an IBM or an IRM), and the mask was subsequently applied to the subband signals of the noisy speech, as illustrated by the dashed line, in order to reconstruct the speech signal.

Fig 1 illustrates the DNN-based system. The AMS feature space was power-compressed with an exponent of 1/15 [17], stacked across frequency channels and fed to the input layer of a feed-forward DNN. The network architecture consisted of an input layer, two hidden layers that each had 128 nodes, and an output layer of 31 nodes. Feature frame concatenation was employed by appending the five past AMS feature time frames to the current frame, which corresponded to a temporal context of 40 ms. The DNN-based system was used to either estimate the IBM or the IRM. The IRM was given by [11]:

$$\text{IRM}(t,f)=(\frac{S^{2}(t,f)}{S^{2}(t,f)+N^{2}(t,f)}{)}^{\beta }=(\frac{\text{SNR}(t,f)}{\text{SNR}(t,f)+1}{)}^{\beta }$$ (1)

In Eq (1), the *S*^2^(*t*, *f*) and the *N*^2^(*t*, *f*) indicate the speech and noise energies, respectively, in a given T-F unit with time frame *t* and frequency channel *f*, and *β* denotes the mask exponent. Mask values in the IRM are therefore scaled according to the SNR, such that T-F units with lower SNR are attenuated more strongly.

### The subband-based system

The subband-based system has previously been employed [8, 14, 21] and a detailed description is given in [14]. In short, delta features were computed symmetrically across frequency bands, resulting in the feature vector **X**(*t*, *f*):

$$\begin{array}{c} \begin{array}{cc} X(t,f) & =[A(t,f),\Delta_{f-k}A(t,f),\Delta_{f+k}A(t,f)]\\ \Delta_{f-k}A(t,f) & =A(t,f)-A(t,f-k),\;\forall k\in \left\{n\in [1;K]|f-n\geq 1\right\}\\ \Delta_{f+k}A(t,f) & =A(t,f)-A(t,f+k),\;\forall k\in \left\{n\in [1;K]|f+n\leq K\right\} \end{array} \end{array}$$ (2)

In Eq (2), *f* indicates the current subband and *k* the considered number of subbands across which the delta features were computed. Seven subbands (*k* = 3) were used in this comparison, since having more than seven subbands does not statistically improve the measured speech intelligibility scores [14]. The classification back-end consisted of a GMM classifier trained to represent the speech and noise-dominated AMS feature distributions (λ_1,*f*_ and λ_0,*f*_) for each subband *f* of the *K* filters [2]. To separate the feature vector into speech- and noise-dominated T-F units, an LC was applied to the *a priori* snr. The GMM classifier output was given as the posterior probability of speech and noise presence *P*(λ_1,*f*_|**X**(*t*, *f*)) and *P*(λ_0,*f*_|**X**(*t*, *f*)), respectively:

$$P(\lambda_{1,f}|X(t,f))=\frac{P(\lambda_{1,f})P(X(t,f)|\lambda_{1,f})}{P(X(t,f))}$$ (3)

$$P(\lambda_{0,f}|X(t,f))=\frac{P(\lambda_{0,f})P(X(t,f)|\lambda_{0,f})}{P(X(t,f))}$$ (4)

The *a priori* probabilities *P*(λ_1,*f*_) and *P*(λ_0,*f*_) were computed by counting the number of feature vectors for each of the classes λ_1,*f*_ and λ_0,*f*_ during training.

### System configurations

In this study, six system configurations were compared (see Table 1). System configurations “GMM (IBM, 1 subband)” and “GMM (IBM, 7 subbands)” exploited spectral context in the subband-based system. In the “GMM (IBM, 1 subband)” configuration, delta features were used as in [2] with only the adjacent subband. In the “GMM (IBM, 7 subbands)” configuration, *k* = 3 symmetrically placed subbands around the considered subband were used to exploit spectral context, according to Eq (2). Configurations “DNN (IBM)”, “DNN (IBM, 40 ms)”, “DNN (IRM)” and “DNN (IRM, 40 ms)” were all configurations of the DNN-based system. “DNN (IBM)” and “DNN (IRM)” were configurations with no frame concatenation and using IBM and IRM estimation, respectively. “DNN (IBM, 40 ms)” and “DNN (IRM, 40 ms)” were configurations with five past concatenated frames corresponding to 40 ms duration, and with IBM and IRM estimation, respectively. In addition to the six system configurations, unprocessed noisy speech was tested as a baseline.

**Table 1:** Overview of the system configurations.

Configuration	Classifier	Architecture	Frame concatenation	Learning objective
GMM (IBM, 1 subband)	GMM	Subband	-	IBM
GMM (IBM, 7 subbands)	GMM	Subband	-	IBM
DNN (IBM)	DNN	Broadband	0 ms	IBM
DNN (IBM, 40 ms)	DNN	Broadband	40 ms	IBM
DNN (IRM, 40 ms)	DNN	Broadband	40 ms	IRM
DNN (IRM)	DNN	Broadband	0 ms	IRM

### Stimuli

The speech material was taken from the Danish Conversational Language Understanding Evaluation (CLUE) database [31]. It consists of 70 sentences in 7 lists for training and 180 sentences in 18 balanced lists for testing, and the sentences are spoken by a male Danish talker. Noisy speech mixtures were created by mixing individual sentences with the non-stationary six-talker (ICRA7) noise [32]. A Long Term Average Spectrum (LTAS) template was computed based on the CLUE corpus, and the LTAS of the noise masker was adjusted to the template LTAS. A randomly-selected noise segment was used for each sentence. In order to avoid onset effects in the speech intelligibility test [31], the noise segment started 1000 ms before the speech onset and ended 600 ms after the speech offset.

### System training and evaluation

The full ICRA7 noise recording of 600 s was divided such that one half of the recording was used for training and the other half was used for testing. The 70 training sentences were each mixed three times with a randomly-selected noise segment from the noise recording at −5, 0, and 5 dB SNR to create a training set of 210 utterances. Training at multiple SNR has been used as an approach in many studies, e.g. [2]. This training set was used to train both the DNN-based system and the subband GMM-based system. The DNN was trained to estimate either the IBM or the IRM using back-propagation with the scaled conjugate gradient algorithm and a mean-squared error cost function. All hidden layers were trained simultaneously in the network. For the IRM estimation, *β* was set to 0.5 as previously done [11, 12]. For the subband GMM-based system, a moderate classifier complexity of 16 Gaussian components with full covariance matrix was selected. The classifiers were first initialized by 15 iterations of the K-means clustering algorithm, followed by five iterations of the expectation-maximization algorithm, and an LC of −5 dB was employed. Both systems were evaluated with 180 CLUE sentences that were each mixed with ICRA7 noise at −5 dB SNR.

### Subjects and experimental setup

The experiment was conducted with a group of 20 NH listeners that were aged between 20 and 32 years with a mean of 24.5 years. Requirements for participation were: (1) aged between 18–40 years, (2) audiometric thresholds of less than or equal to 20 dB hearing level (HL) in both ears (between 0.125 and 8 kHz), (3) Danish as their native language, and (4) no previous experience with the Hearing In Noise Test (HINT) [33] or CLUE material [31].

The total session lasted about two hours, including the screening process. The experiment was approved by the Danish Science-Ethics Committee (reference H-16036391). Listeners were recruited with online advertisement, and they were paid for their participation. Informed consent was obtained prior to the experiment. The subjects were all recruited and tested within a two-month period. The experiment was split into two parts: subject training and subject testing. In the training part, five randomly selected sentences from the training set were presented for each of the conditions to familiarize the subjects with the task. Subsequently, each subject heard one list per condition, whereby conditions and lists were randomized across subjects. The sentences were presented diotically to the listener via headphones (Sennheiser HD650) in an acoustically and electrically shielded booth. Prior to the actual experiments, the headphones were calibrated by first adjusting to a reference sound pressure level (SPL) and then performing a headphone frequency response equalization. During the experiment, the sentences were adjusted to the desired presentation level, and the equalization filters were applied. The SPL was set to a level of 65 dB. For each sentence, the subjects were instructed to repeat the words they heard, and an operator scored the correctly understood words via a Matlab interface. The subjects were told that guessing was allowed. They could listen to each sentence only once, and breaks were allowed according to the subject’s preference.

### Statistical analysis

Intelligibility scores were reported as a percentage of correctly scored words, i.e. the word recognition score (WRS). The WRSs were computed per sentence and averaged across sentences per list. The intelligibility scores followed a normal distribution, and a linear mixed effect model was constructed with list WRSs as the response variable and the system configurations as a fixed factor (8 levels). Subjects were treated as a random factor, as is standard in a repeated measures design. Fixed factor levels were tested at a 5% significance level. To visualize the data, the predicted least-squares means and 95% confidence limits of the least-squares means were extracted from the model. To assess any difference between system configurations, the differences of the least-squares means were computed in pairwise comparisons, where the *p* values were adjusted following the Tukey multiple comparison testing. To evaluate potential speech intelligibility improvements, Paired Student’s *t*-tests between the noisy speech and the relevant system configuration was constructed and tested at a 5% significance level.

## Results

**Fig 2:**
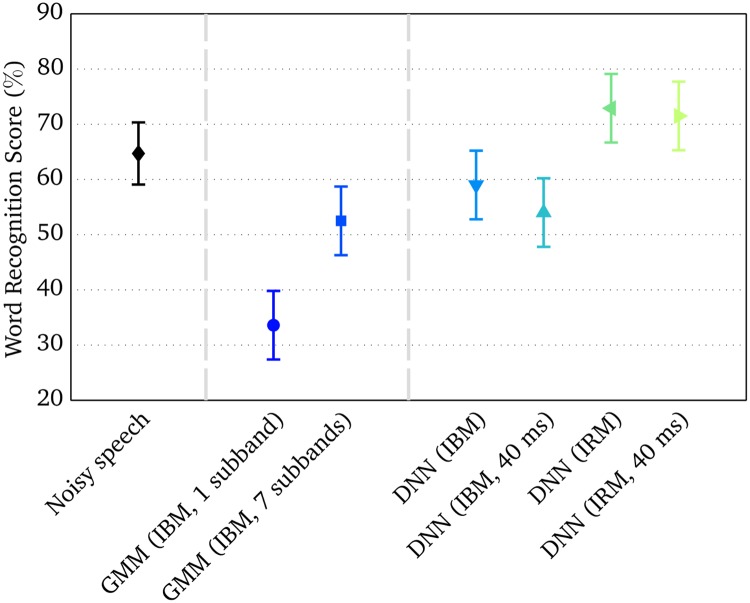
Measured WRSs in normal-hearing listeners at −5 dB SNR in the ICRA7 noise. Unprocessed noisy speech served as a baseline condition. For the baseline (diamonds), sample means across subjects and 95% Student’s *t*-based confidence intervals of the mean were computed. For the system configurations, the least-squares means and 95% confidence limits of the least-squares means predictions derived from the linear mixed effect model were plotted.

Fig 2 shows the measured WRSs of the six system configurations along with unprocessed noisy speech. The sample mean across subjects and a 95% Student’s *t*-based confidence interval of the sample mean were computed and included in Fig 2 for visualization. For the six system configurations, the least-squares means and 95% confidence limits of the least-squares means predictions are shown. In noisy speech, the average WRS was 65%. The relatively high baseline score was presumably due to the fluctuations in the six-talker noise, which has been shown to facilitate listening-in-the-dips in NH subjects [34].

Measured WRSs increased significantly from the “GMM (IBM, 1 subband)” configuration to the “GMM (IBM, 7 subbands)” configuration by 18.9 percentage points (*p* < 0.0001). This result indicates that an increased number of appended delta feature vectors across frequency in the subband GMM-based system led to higher measured speech intelligibility, since a larger amount of spectral context was exploited. Comparing across the systems, the “DNN (IBM)” configuration led to 25.4 percentage points higher WRS than the “GMM (IBM, 1 subband)” configuration (*p* < 0.0001). Despite that the “DNN (IBM)” configuration had a higher WRS of 6.5 percentage points than the “GMM (IBM, 7 subbands)” configuration, measured speech intelligibility scores for the two configurations were not significantly different.

The “DNN (IBM)” and “DNN (IBM, 40 ms)” configurations did not differ significantly from each other, and no statistically significant difference was found either between the “DNN (IRM)” and “DNN (IRM, 40 ms)” configurations. Therefore, the employed time frame concatenation technique, which was used to exploit temporal context, did not have a perceptual effect in the current DNN-based system, regardless of whether IBM or IRM estimation was considered in the system.

The configuration “DNN (IRM)” led to 13.9 percentage points higher WRS than the “DNN (IBM)” configuration (*p* < 0.001). Furthermore, 17.5 percentage points higher WRS was observed for the “DNN (IRM, 40 ms)” configuration than for the “DNN (IBM, 40 ms)” configuration (*p* < 0.0001). Therefore, a clear perceptual advantage was found for IRM over IBM estimation in the DNN-based system. The measured intelligibility scores were subsequently converted into WRS improvements relative to the unprocessed noisy speech. Significant improvements, based on the Paired Student’s *t*-tests at a 5% significance level, were obtained for the “DNN (IRM)” configuration (8.2 percentage points; *t*[19] = 2.36; *p* = 0.014) and the “DNN (IRM, 40 ms)” configuration (6.8 percentage points; *t*[19] = 2.14; *p* = 0.023). This particular finding demonstrates the benefit of estimating the IRM as opposed to the IBM, when computational speech segregation systems are used for noise reduction applications.

## Discussion

### The roles and relative contributions of the components

**Fig 3:**
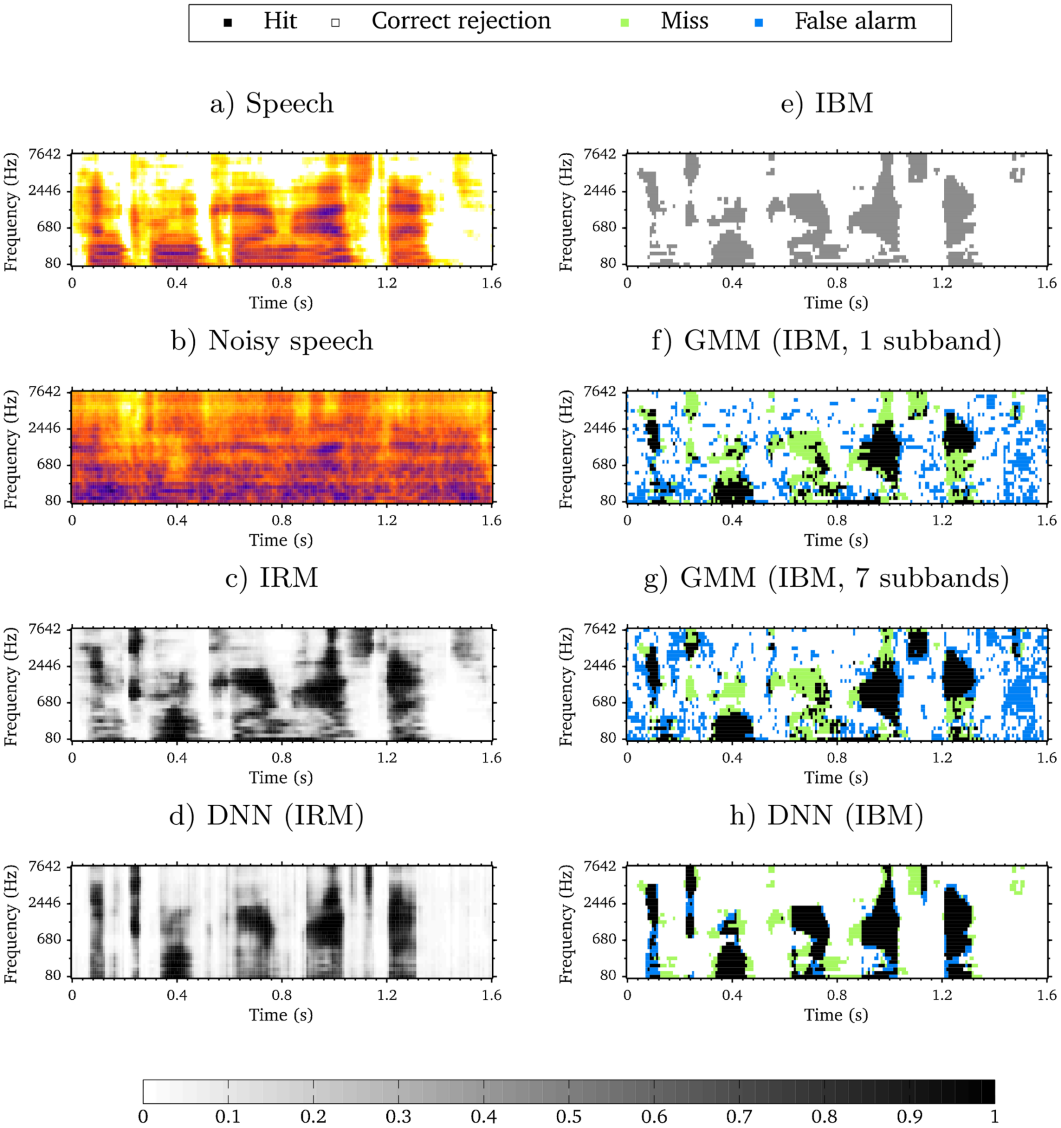
Estimated and ideal time-frequency masks. Masks for an CLUE sentence mixed with ICRA7 noise at −5 dB SNR. The spectrograms of clean and noisy speech are shown in Figs 3a and 3b. The IRM and the IBM are shown in Figs 3c and 3e. A selection of estimated masks from system configurations are shown in Figs 3d, 3f, 3g and 3h. Misses (speech-dominated T-F units erroneously labeled as noise-dominated) and false alarms (noise-dominated T-F units erroneously labeled as speech-dominated) are shown on top of the estimated IBMs. The estimated IBM in Fig 3h was converted from the corresponding estimated IRM by applying a threshold, which was derived from Eq (1) at −5 dB SNR and using *β* = 0.5.

The comparison between the subband GMM-based system configurations (“GMM (IBM, 1 subband)” and “GMM (IBM, 7 subbands)”) indicated that the measured speech intelligibility scores increased with the number of subbands used to compute the delta features. By increasing the number of subbands, the AMS feature vector was rapidly growing. In [14], it was shown that more than seven considered subbands did not further increase the measured speech intelligibility. The subband GMM classifier was therefore limited in the capability to handle the large amount of AMS feature data. In addition, the “GMM (IBM, 1 subband)” configuration that resembled previously-used approaches [2, 8, 21] resulted in a much lower speech intelligibility than the corresponding broadband DNN-based system configuration (“DNN (IBM)”). By increasing the number of subbands and thereby exploiting more spectral context in the subband GMM-based system, it was possible to achieve a measured speech intelligibility score similar to that obtained with the DNN-based system. By changing the architecture from subband GMM classifiers to a broadband DNN, the segregation system was able to predict the T-F units simultaneously across all of the subbands. Therefore, the DNN-based system exploited the spectral context in a broadband manner, which may be more effective than the corresponding subband-based system. This is most likely because of the capability of DNNs to handle higher-dimensional feature vectors. Estimated IBMs with these three configurations (“GMM (IBM, 1 subband)”, “GMM (IBM, 7 subbands)” and “DNN (IBM)”) are shown in Fig 3f–3h and can be compared to the IBM in Fig 3e. H-FA rates were computed for each of the estimated IBMs to indicate the mask estimation accuracy. Results were 27.8% (“GMM (IBM, 1 subband)”), 34.5% (“GMM (IBM, 7 subbands)”) and 63.7% (“DNN (IBM)”), respectively. A larger amount of spectral context is exploited by increasing the number of considered subbands in the subband GMM-based system (Fig 3f and 3g), which leads to more correctly-classified speech T-F units (hits) and therefore a larger H-FA rate. However, the estimated IBM using the DNN-based system (Fig 3h) contains much larger regions with correctly-classified speech T-F units and and less mask errors (both misses and false alarms), which has increased the H-FA rate quite substantially. The results of the present study also indicated that the employed time frame concatenation technique, which has been proposed to exploit temporal context in the state-of-the-art approaches [11, 12, 17], did not have a significant impact on the measured speech intelligibility. This was observed regardless of whether the DNN-based system estimated the IBM or the IRM. This result was rather surprising, but should be seen in light of the small amount of training data (only 210 utterances) fed to the DNN-based system. Most likely, the small amount of training data was not sufficient to unfold the predictive power of the DNN. Another important point is that “only” five past feature frames were appended to the current frame, resulting in an exploited temporal context of 40 ms. To put this into perspective, 23 frames were concatenated in total with a step size of 10 ms in another study [17], which resulted in a much larger exploited temporal context of 200 ms. Furthermore, the 23 frames were symmetrically placed around the current frame with eleven past and eleven future time frames. Whether the temporal context in future time frames affect speech intelligibility is not clear.

A substantial perceptual advantage of IRM over IBM estimation was observed in the DNN-based system, where both configurations with IRM estimation (“DNN (IRM)” and “DNN (IRM, 40 ms)”) led to higher measured speech intelligibility scores than the corresponding configurations with IBM estimation. The present study therefore demonstrated the effectiveness of the IRM estimation over the IBM estimation with respect to measured speech intelligibility in the state-of-the-art approaches. The effectiveness of the IRM can be explained by how the mask gain values are computed. From Eq (1), it is observed that these values can vary continuously between 0 and 1. Comparing the ideal masks (Fig 3c and 3e) to the spectrogram of speech in quiet (Fig 3a), it can be seen that several mask regions with low speech energy are captured by the IRM, but not by the IBM (e.g., around 0.6 s and above 2446 Hz). The IRM can therefore convey important speech information that is not reflected in the IBM, suggesting that the IRM is a better learning objective than the IBM in computational speech segregation. By comparing the estimated masks in Fig 3d and 3h, it is also apparent that the estimated IRM mask values are more tolerant to misses by the segregation system. Several mask regions with misses in Fig 3h correspond to areas with positive gain values in Fig 3d, such that speech information is conveyed, which otherwise would have been missed. Therefore, even though a binary classification of T-F units makes the IBM a simpler objective to estimate, the findings in the present study support the use of the IRM estimation in state-of-the-art approaches for noise reduction applications. In addition to the measured speech intelligibility, subjective speech quality will most likely also improve with IRM estimation, since it has previously been demonstrated that the IRM itself improves the quality in comparison to the IBM [26].

Finally, the relative contributions of the components within state-of-the-art approaches were addressed. First, a substantial improvement of 25.4 percentage points in measured speech intelligibility scores was found by changing the system architecture from subband GMM-based, with first-order delta features across frequency, to the broadband DNN architecture. The subband GMM-based architecture was similar to previously-used system architectures [2, 8, 21]. Secondly, by changing from IBM estimation to IRM estimation, another improvement of 13.9 percentage points in measured speech intelligibility scores was obtained. Therefore, these results suggest that both of these components play a significant role in the success of the state-of-the-art approaches. By combining the two significant components, intelligibility improvements of about 7–8 percentage points relative to noisy speech were demonstrated. These improvements were obtained despite that the system was evaluated in the challenging scenario of being presented with unseen, six-talker noise at a low SNR after a relatively limited system training.

### Large-scale training in the DNN-based system

Being able to generalize to acoustic conditions not seen during training (i.e., mismatches between acoustic conditions encountered during training and testing) is crucial for any speech segregation system to be applied in realistic scenarios. The segregation systems in this study considered a mismatch of six-talker noise segments between training and testing. One reason for the relatively limited speech intelligiblity improvement with the DNN-based system with IRM estimation, in comparison to that which has been reported in other studies, is that the competing six-talker noise contains spectro-temporal modulations that are very similar to the modulations in the speech signal. This complicates the task of automatically segregating the interfering noise from the target speech. Other studies have demonstrated a generalization ability with DNN-based systems but have employed 20-talker noise with less fluctuations [16, 17].

Another reason for the limited improvement is the small amount of training data used in the present study. The training set was kept low with only 210 utterances in order to compare the DNN-based system with the subband GMM-based system. However, it has previously been shown that DNNs can benefit from large-scale training in computational speech segregation [17, 22, 35], and intelligibility improvements over noisy speech can be obtained with these systems in conditions with various acoustic mismatches [16–19]. In one of these studies [16], the speech segregation system was trained with 28, 000 utterances presented in different types of noise at different SNRs. At −5 dB SNR and with 20-talker noise, this led to an improvement of 25 percentage points in speech intelligibility scores in NH listeners. In another study [17], the system was trained with 640, 000 utterances in a multi-conditional training set to produce an improvement of 10 percentage points in the speech intelligibility scores in the same experimental design as the first study [16]. Retraining the considered DNN-based system with a larger training set than 210 utterances would most likely improve the generalization ability to the unseen six-talker noise segments. Large-scale training is therefore also an important component within state-of-the-art approaches in computational speech segregation, and investigating the impact of large-scale training on measured speech intelligibility is one direction for future work.

## Conclusion

This study explored the relative contributions of a selection of components within state-of-the-art speech segregation systems to improving speech intelligibility. The first component was the system architecture, which was changed from subband-based, in which a classifier was employed per frequency channel, to a DNN network architecture where the T-F units were predicted simultaneously across all frequency channels. Specifically, a broadband DNN-based system was compared with a corresponding subband GMM-based system. A second component was the time frame concatenation technique. This technique is often applied in DNN-based speech segregation systems to exploit the temporal context. However, this technique did not show a significant effect on the measured speech intelligibility scores in this study, presumable because of the relatively limited amount of training data was not sufficient to unfold the predictive power of the DNN. The third considered component was the estimation of the IRM instead of estimating the IBM. Results showed a substantial perceptual advantage with the IRM estimation in the DNN-based system. Finally, the relative contributions of the components were addressed. A substantial improvement of 25.4 percentage points in measured speech intelligibility scores was found by changing the system architecture from subband GMM-based, which is similar to previously-used architectures, to a recent DNN architecture. Another improvement of 13.9 percentage points was obtained by changing from IBM estimation to IRM estimation in the state-of-the-art approach. Therefore, both of these components seem to play a significant role in the success of state-of-the-art speech segregation systems. By combining the two significant components, intelligibility improvements of about 7–8 percentage points relative to noisy speech were demonstrated in adverse conditions where speech was corrupted by a six-talker noise at a low SNR.
